# Poor prognosis of single hormone receptor- positive breast cancer: similar outcome as triple-negative breast cancer

**DOI:** 10.1186/s12885-015-1121-4

**Published:** 2015-03-18

**Authors:** Soo Youn Bae, Sangmin Kim, Jun Ho Lee, Hyun-chul Lee, Se Kyung Lee, Won Ho Kil, Seok Won Kim, Jeong Eon Lee, Seok Jin Nam

**Affiliations:** Department of Surgery, Samsung Medical Center, Sungkyunkwan University School of Medicine, 50 Irwon-dong, Kangnam-gu, 135-710 Seoul, South Korea

**Keywords:** Breast cancer, Estrogen receptor, Progesterone receptor, Human epidermal growth factor receptor 2, Prognosis

## Abstract

**Background:**

Response to endocrine therapy in breast cancer correlates with estrogen receptor (ER) and progesterone receptor (PR) status. Generally, hormone receptor-positive (HR+) breast cancers have favorable prognosis. In order to understand the exact clinical characteristics and prognosis of single HR-positive breast cancer (ER + PR- tumors and ER-PR+ tumors), we compared these tumors to double HR+ tumors as well as HR- negative tumors (ER-PR-).

**Methods:**

We examined the clinical and biological features of 6,980 women with invasive ductal carcinoma, and these patients were stratified according to ER and PR expression as double HR+ (ER + PR+), single HR+ (ER + PR- and ER-PR+) and double HR-negative (HR-, ER-PR-) tumors.

**Results:**

In this study, 571 (8.2%) cases were single HR+ tumors, of which 90 (1.3%) were ER-PR+ tumors and 481 (6.9%) were ER + PR- tumors. Our multivariate analysis showed that in patients without HER2 overexpression ER + PR- tumors were associated with an increased risk of recurrence and death compared with ER + PR+ tumors, with a hazard ratio of 2.12 for disease-free survival (DFS) and 4.79 for overall survival (OS). In patients without HER2 overexpression ER-PR+ tumors had increased risk of recurrence and death compared with ER + PR+ tumor, with a hazard ratio of 4.19 for DFS and 7.22 for OS. In contrast, in patients with HER2 overexpression, the difference in survival between single HR+ tumors and double HR+ HR- tumors was not statistically significant. In patients without HER2 overexpression the DFS and OS of ER + PR- and ER-PR+ tumors were not significantly different from those of ER-PR- tumors.

**Conclusion:**

We have identified clinically and biologically distinct features of single HR+ tumors (ER–PR+ and ER + PR–) through comparison with both ER + PR+ and ER-PR- tumors. These differences were only significant in HER2- tumors, not in HER2+ tumors. Single HR+ tumors without HER2 overexpression (ER + PR-HER2- or ER-PR + HER2-) were associated with poorer survival than ER + PR + HER2- tumors, and had comparable poor survival to ER-PR-HER2- tumors (triple-negative breast cancer).

**Electronic supplementary material:**

The online version of this article (doi:10.1186/s12885-015-1121-4) contains supplementary material, which is available to authorized users.

## Background

In breast cancer, steroid hormone receptors (HRs; i.e., estrogen receptor [ER] or progesterone receptor [PR]) have been shown to be important prognostic factors and predictive markers for response to endocrine therapy in the treatment of breast cancer. About 70% of breast cancers are hormone receptor-positive tumors (HR+). HR+ breast cancers generally have a favorable prognosis, but HR-negative (HR-) breast cancers have a poor prognosis. PR is an estrogen-regulated gene; ER-positive (ER+) tumors are usually also PR positive (PR+), whereas ER-negative (ER-) tumors are usually PR negative (PR-). Therefore, single HR+ (i.e., ER+/PR- or ER-/PR+) tumors represent a minority of breast cancers.

Clinical data have shown in both the metastatic and adjuvant treatment settings that tamoxifen is less efficacious in ER + PR− tumors than in ER + PR+ tumors [1-3], and single HR+ breast cancers, especially ER + PR- breast cancers, have aggressive features and poorer prognosis in comparison to double HR+ (ER + PR+) breast cancer [4,5]. However, to our knowledge, comparative studies of HR- (ER-PR-) breast cancers are very limited [6,7].

Previous studies have shown that ER + PR- tumors exhibit high expression of epidermal growth factor receptors [1,4,7-11], but in most studies, the prognosis of ER + PR- tumors was determined without considering human epidermal growth factor receptor 2 (HER2) expression. Moreover, these studies had a common limitation, in that prognosis was evaluated without considering trastuzumab treatment.

Previous studies have suggested that ER-PR+ tumors have poorer prognosis than ER + PR+ tumors [10,12-16]. However, due to the rarity of ER-PR+ breast cancer (a reported incidence of 1.5-3.4% [10,12-15]), the characteristics and prognosis of this tumor are not well known.

Therefore, in order to understand the exact clinical characteristics and prognosis of single HR-positive breast cancer (ER + PR- tumors and ER-PR+ tumors), we compared these tumors to double HR+ tumors as well as HR- tumors (ER-PR-), and stratified these results according to HER2 overexpression.

## Methods

Patients were selected from the clinical database of the Breast Cancer Center at Samsung Medical Center, Korea, between January 2003 and July 2013. A total of 7,010 women with invasive ductal carcinoma were identified. Of them, 6,980 patients were selected for this study excluding patients who were diagnosed with bilateral tumors or with distant metastases at preoperative work-up or underwent neoadjuvant chemotherapy.

We reviewed the clinicopathologic characteristics of patients, including biologic factors, such as ER, PR, HER2, epidermal growth factor receptors (EGFR), and Ki-67. The pathologic tumor stage was assessed according to the American Joint Committee on Cancer (AJCC) 6th Staging System. For ER and PR staging, nuclear (not cytoplasmic) staining was scored using the Allred score (AS) interpretation system, a method that provides semi-quantitative measurement of the proportion of positive cells (scored on a 0 to 5 scale) and staining intensity (scored on a 0 to 3 scale), with a maximum score of 8; an AS > 2 considered positive.

HER2 positivity was defined as an intensity of 3+ by IHC, a score of 2+ was interpreted as equivocal. A negative test was defined as staining with a score of 0/1+. For equivocal stating, silver in situ hybridization (SISH) or fluorescence in situ hybridization (FISH) were performed; the results were positive for HER2 amplification when the ratio of HER2 to CEP17 was > 2.2.

For EGFR and/or Ki-67, results were considered positive based on identification of the following criteria in at least one core. Immunostaining for EGFR was interpreted as positive when at least 10% of the tumor cells showed moderate to strong membrane staining. Ki-67 was considered positive when ≥ 14.0% of cells showed staining [17].

Differences in the frequencies of clinicopathological factors and subtypes were statistically analyzed using the chi-square test and Fisher’s exact test. Disease-free survival (DFS) was defined as the time from surgery to the date of documentation of relapse, including locoregional recurrence and/or distant metastasis. Overall survival (OS) was defined as the number of months from surgery to the date of death. Survival curves were constructed using the Kaplan-Meier method. Hazard ratios were estimated using a Cox regression for DFS/OS in a multivariate analysis. Statistical significance was defined as *P* < 0.05. All statistical analyses were performed using SPSS Statistics 21.0 (IBM).

Study data were collected using a protocol approved by the Institutional Review Board of Samsung Medical Center, Korea (IRB number 2014-09-111). Specific patient consent was not required because we used retrospective data from medical records of patients who had previously signed information release documents.

## Results and discussion

### Clinicopathologic characteristics of single hormone receptor- positive breast cancer

The median follow-up duration for the 6,980 patients included in this analysis was 45 months (range, 1-133 months). In this study, 4,651 (66.6%) cases were double HR+ (ER + PR+) tumors, 1,758 (25.2%) were double HR- (ER-PR-) tumors, and 571 (8.2%) cases were single hormone-receptor positive tumors, of which 90 (1.3%) cases were ER-PR+ tumors and 481 (6.9%) were ER + PR- tumors. The clinicopathological characteristics of the four subtypes are summarized in Table 1. Overall, ER+/PR- tumors were found more frequently in postmenopausal women (61.5%) than other subtypes (P < 0.001). Compared with ER + PR+ tumor, ER + PR- tumors were not significantly different in staging (P = 0.083), but ER + PR- tumors exhibited higher nuclear grade (NG,P <0.001), higher Ki-67 level (Ki-67 ≥ 14.0, 76.0% vs. 53.9%, P < 0.001), and higher EGFR and HER2 expression (p < 0.001). However, compared with ER-PR- tumors, ER + PR- tumors showed lower stage (stage I, ER + PR- 43.7% vs. ER-PR- 35.8%, P = 0.027), lower NG (P < 0.001), lower Ki-67 level (P < 0.001), lower p53 expression (P <0.001) and lower EGFR expression (P < 0.001), but there was no difference in HER2 overexpression (P = 0.089).

**Table 1:** **Clinicopathologic characteristics of patients with ER + PR+, ER + PR-, ER-PR+ and ER-PR- tumors**

	ER + PR+ (N = 4651)		ER-PR+ (N = 90)		ER-PR- (N = 1758)		ER + PR- (N = 481)	
Age, median (range)	47 (20-90)		48 (22-72)		49 (21-85)		54 (27-84)	
Menopause								
Postmenopause	1489	(32.4%)	34	38.6%	836	(48.4%)	319	(67.3%)
Premenopause	3110	(67.6%)	54	61.4%	893	(51.6%)	155	(32.7%)
Uknown	52		2		29		7	
Operation								
MRM	1363	(29.3%)	40	(44.4%)	585	(33.3%)	171	(35.6%)
BCS	3288	(70.7%)	50	(55.6%)	1173	(66.7%)	310	(64.4%)
pT								
T1	2985	(64.2%)	49	(54.4%)	860	(48.9%)	286	(59.5%)
T2	1472	(31.6%)	38	(42.2%)	835	(47.5%)	184	(38.3%)
T3	184	(4.0%)	3	(3.3%)	62	(3.5%)	10	(2.1%)
T4	10	(0.2%)	0	(0.0%)	1	(0.1%)	1	(0.2%)
pN								
N0	2717	(58.4%)	48	(53.3%)	1105	(62.9%)	303	(63.0%)
N1	1368	(29.4%)	29	(32.2%)	445	(25.3%)	129	(26.8%)
N2	363	(7.8%)	8	(8.9%)	133	(7.6%)	32	(6.7%)
N3	203	(4.4%)	5	(5.6%)	75	(4.3%)	17	(3.5%)
Stage								
I	2156	(46.4%)	32	(35.6%)	630	(35.8%)	210	(43.7%)
IIA	1266	(27.2%)	30	(33.3%)	635	(36.1%)	162	(33.7%)
IIB	605	(13.0%)	14	(15.6%)	266	(15.1%)	54	(11.2%)
IIIA	413	(8.9%)	9	(10.0%)	151	(8.6%)	37	(7.7%)
IIIB	8	(0.2%)	0		1	(0.1%)	1	(0.2%)
IIIC	203	(4.4%)	5	(5.6%)	75	(4.3%)	17	(3.5%)
Nuclear Grade								
I	974	(21.0%)	1	(1.1%)	12	(0.7%)	67	(14.0%)
II	2656	(57.3%)	26	(29.2%)	358	(20.4%)	206	(43.1%)
III	1006	(21.7%)	62	(69.7%)	1383	(78.9%)	205	(42.9%)
unknown	15		1		5		3	
HER2								
Positive	518	(11.4%)	30	(34.5%)	671	(38.8%)	159	(34.5%)
Negative	4018	(88.6%)	57	(65.5%)	1058	(61.2%)	302	(65.5%)
Unknown	115		3		29		20	
Ki-67								
≥ 14.0%	2202	(53.9%)	56	(91.8%)	1334	(93.9%)	292	(76.0%)
< 14.0%	1887	(46.1%)	5	(8.2%)	87	(6.1%)	92	(24.0%)
Unknown	562		29		337		97	
p53								
Positive	1008	(21.8%)	49	(55.1%)	1021	(58.4%)	162	(34.2%)
Negative	3622	(78.2%)	40	(44.9%)	727	(41.6%)	311	(65.8%)
Unknown	21		1		10		8	
Chemotherapy								
Yes	3127	(68.7%)	78	(89.7%)	1575	(91.9%)	345	(73.7%)
No	1424	(31.3%)	9	(10.3%)	138	(8.1%)	123	(26.3%)
Unknown	100		3		45		13	
Radiotherapy								
Yes	3543	(78.0%)	55	(64.0%)	1258	(73.8%)	334	(70.9%)
No	1001	(22.0%)	31	(36.0%)	446	(26.2%)	137	(29.1%)
Unknown	107		4		54		10	
Endocrine Therapy								
Yes	4490	(99.2%)	75	(88.2%)	2	(0.1%)	454	(97.0%)
No	34	(0.8%)	10	(11.8%)	1704	(99.9%)	14	(3.0%)
Unknown	127		5		52		13	

ER-PR+ tumors had higher NG (P <0.001), higher Ki-67 level (P < 0.001), and higher expression of p53 and EGFR (P < 0.001) than ER + PR+ tumors. However, compared with ER-PR- tumors, there was no difference in stage (P = 0.979) or NG (P = 0.0117). Also, there was no difference in expression of Ki-67 (P = 0.511), p53 (P = 0.531), EGFR (P = 0.055) or HER2 (P = 0.419).

Both ER-PR+ and ER + PR- tumors were shown to have higher HER2 overexpression (34.5%) than ER + PR+ tumors (11.4%, P < 0.001), but had similar HER2 overexpression to ER-PR- tumors (38.8%, P = 0.192). The characteristics of single hormone receptor-positive (ER + PR- and ER-PR+) tumors were more distinct in HER2-negative (HER2-) tumors than in HER2 overexpressing (HER2+) tumors. (Additional file 1: Table S1 and Table S2).

### Survival analysis of single hormone receptor- positive breast cancer

Approximately 97% of patients with ER + PR- tumors and 88% of patients with ER-PR+ tumors received endocrine therapy. More patients with ER + PR- (73.7%) and ER-PR+ (89.7%) tumors received chemotherapy than the group with ER + PR+ tumors (68.7%), but less than the group with ER-PR- tumors (91.9%, Table 1). Approximately 72% of patients with ER + PR- tumors received both endocrine therapy and chemotherapy, and 24.9% of patients received only endocrine therapy. In ER-PR+ tumors, 80% of patients received both chemotherapy and endocrine therapy, 8.2% of patients received only endocrine therapy and 9.4% of patients received only chemotherapy.

**Figure 1:**
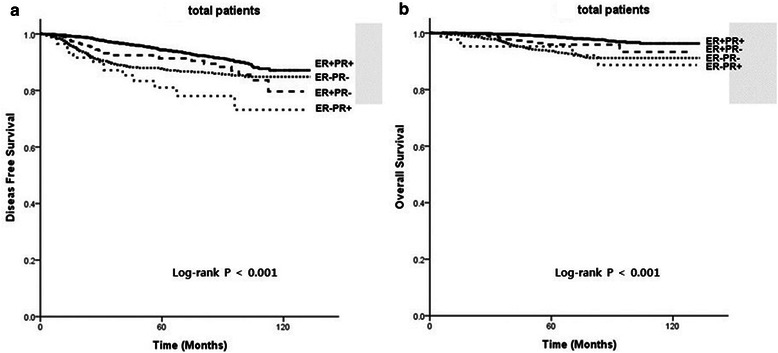
**(a) Disease-free survival (DFS) and (b) overall survival (OS) of all patients.**

With univariate analysis by Kaplan-Meier method, the survival graph of ER + PR- tumors was located between that of ER + PR+ tumors and ER-PR- tumors. The 5-year and 10-year DFS of ER + PR- tumors was 91.4% and 79.6%, respectively, and the 5-year and 10-year OS was 95.9% and 93.9%, respectively. Patients with ER-PR+ tumors had worse DFS (5-year 81.0%; 10 year 73.1%) and OS (5-year 95.3%; 10-year 88.7%, Figure 1) than those with ER + PR-.

**Figure 2:**
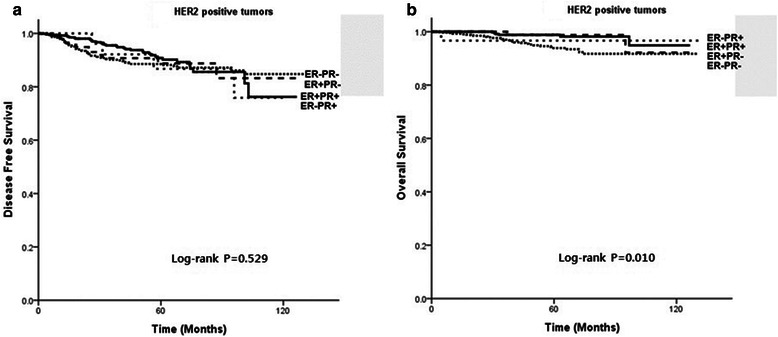
**(a) Disease-free survival (DFS) and (b) overall survival (OS) of patients with HER2-positive tumors.**

Among 1,376 patients with HER2 overexpression, there was no significant difference in DFS between four subgroups (P = 0.529), and patients with ER-PR-HER+ tumors had the worst OS (P = 0.010, Figure 2). However, the 790 patients who received trastuzumab therapy had similar OS (P = 0.113), as did the 586 patients who did not receive trastuzumab therapy (P = 0.147).

**Figure 3:**
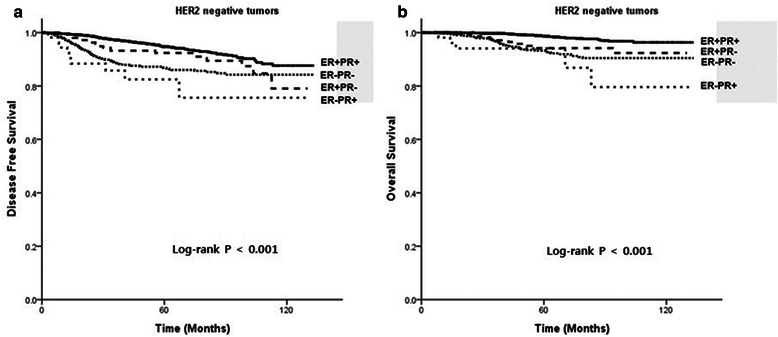
**(a) Disease-free survival (DFS) and (b) overall survival (OS) of patients with HER2-negative tumors.**

In 5,433 patients without HER2 overexpression, ER + PR- tumors were associated with poorer OS than ER + PR+ tumors (P < 0.001), but similar OS to ER-PR- tumors (P =0.338). ER-PR+ tumors also had poorer OS than ER + PR+ tumors (P < 0.001), but there was no significant difference from the OS of ER-PR- tumors (P = 0.165, Figure 3).

With multivariate analysis, in patients with HER2 overexpression, the single HR+ (ER + PR- and ER-PR+) tumors seem to increase risk of recurrence, but this difference was not significant (Table 2). In patients without HER2 overexpression, ER + PR- tumors had increased risk of recurrence and death compared with ER + PR+ tumors, with a hazard ratio of 2.12 (95% CI 1.20 -3.75) for DFS and 4.79 (95% CI 1.84-12.18) for OS. ER-PR+ tumors were at increased risk of recurrence and death compared with ER + PR+ tumors, with a hazard ratio of 4.19 (95% CI; 1.86-10.02) for DFS and 7.22 (95% CI 1.62-32.06) for OS (Table 3). ER + PR- tumors and ER-PR+ tumors were not significantly different in terms of DFS and OS compared ER-PR- tumors.

**Table 2:** **Multivariate analysis of disease-free survival (DFS) and overall survival (OS) in 1.376 women with HER2-positive breast cancer**

		B coefficients	Standard error	Wald	*P*	Hazard ratio	95.0% confidence interval	
							Lower	Upper
DFS	ER + PR+ vs. ER-PR+	.391	.619	.399	0.528	1.478	.439	4.973
	ER + PR+ vs. ER-PR-	.394	.261	2.284	0.131	1.483	.890	2.471
	ER + PR+ vs. ER + PR-	.457	.388	1.389	0.239	1.580	.738	3.382
	ER-PR- vs. ER-PR+	-.003	.619	.000	0.996	.997	.296	3.357
	ER-PR- vs. ER + PR-	.064	.373	.029	0.864	1.066	.513	2.214
OS	ER + PR+ vs. ER-PR+	−7.587	82.200	.009	0.926	.001	.000	4.71E + 66
	ER + PR+ vs. ER-PR-	1.376	.583	5.576	0.018	3.958	1.263	12.398
	ER + PR+ vs. ER + PR-	.241	1.125	.046	0.830	1.273	.140	11.549
	ER-PR- vs. ER-PR+	−8.962	82.199	.012	0.913	.000	.000	1.18E + 66
	ER-PR- vs. ER + PR-	−1.135	1.036	1.200	0.273	.322	.042	2.449

(Adjusted for age, stage, nuclear grade, Ki-67and trastuzumab treatment).

**Table 3:** **Multivariate analysis of disease-free survival (DFS) and overall survival (OS) in 5,433 women with HER2-negtive breast cancer**

		B coefficients	Standard error	Wald	*P*	Hazard ratio	95.0% confidence interval	
							Lower	Upper
DFS	ER + PR+ vs. ER-PR+	1.463	.429	11.608	0.001	4.319	1.861	10.020
	ER + PR+ vs. ER-PR-	.913	.170	28.982	0.000	2.493	1.788	3.476
	ER + PR+ vs. ER + PR-	.753	.291	6.702	0.010	2.123	1.201	3.755
	ER-PR- vs. ER-PR+	.550	.422	1.695	0.193	1.733	.757	3.963
	ER-PR- vs. ER + PR-	-.160	.296	.293	0.589	.852	.477	1.523
OS	ER + PR+ vs. ER-PR+	1.977	.761	6.755	0.009	7.220	1.626	32.061
	ER + PR+ vs. ER-PR-	1.774	.307	33.488	0.000	5.895	3.232	10.751
	ER + PR+ vs. ER + PR-	1.564	.478	10.722	0.001	4.779	1.874	12.189
	ER-PR- vs. ER-PR+	.203	.728	.078	0.781	1.225	.294	5.105
	ER-PR- vs. ER + PR-	-.210	.462	.207	0.649	.811	.328	2.004

(Adjusted for age, stage, nuclear grade and Ki-67).

## Discussion

We have evaluated in detail the biological characteristics and prognosis of single HR+ tumors through comparison with ER + PR+ tumors as well as ER-PR- tumors. In our series, 8.2% of cases were ER + PR- and 1.4% were ER-PR+. These numbers are somewhat smaller than those from previously published series where 10-15% of cases were ER + PR- and 2-4% were ER-PR+. Most previous studies included patients with breast cancer regardless of histologic type, but we analyzed patients with invasive ductal carcinoma [10,12,13,18]. However, the clinical and biological features of ER + PR- tumors were consistent with those found in previous studies, and there was a high incidence in postmenopausal women. In terms of NG and IHC of Ki-67 level, p53 and EGFR, ER + PR- tumors showed moderate characteristics between the levels of ER + PR+ and ER-PR- tumors, while ER-PR+ tumors were more similar to ER-PR- tumors than ER + PR+ tumors. In addition, on Kaplan-Meier analysis, the survival graph of the ER + PR- tumors was located in between those of ER + PR+ tumors and ER-PR- tumors, and ER-PR+ tumors were shown to have worse survival than ER-PR- tumors.

In previous studies, loss of PR has been suggested to be a marker of aberrant growth factor signaling and has been associated with one mechanism for endocrine resistance[11], and several studies have shown that ER + PR- tumors exhibit high expression of epidermal growth factor receptors [1,4,7-11]. In our cases, HER2 overexpression was 34.5% in single HR+ tumors, and as high as 38.8% in ER-PR- tumors, but the rate of HER2 overexpression in ER + PR+ tumors was 11.4%. Therefore, we stratified our cases according to HER2 overexpression and we found that differences in clinicopathologic characteristics were not significantly different between the four subgroups (ER + PR+, ER-PR+, ER-PR- and ER + PR-) in patients with HER2 overexpression. In addition, there was no difference in survival between these four subgroups. However, in patients without HER2 overexpression, significant differences in biological characteristics were shown more distinctly; ER + PR-, ER-PR+ tumors and ER-PR-HER2- tumors (triple-negative breast cancer, TNBC) were both associated with poor survival.

As demonstrated in previous studies, PR negativity may be association with cross talk with epidermal growth factor receptor- i.e., HER2 or EGFR. In our study, ER + PR- tumors showed high HER2 overexpression. Nevertheless, PR negativity was not a significant prognostic factor in tumors with HER2 overexpression. This suggests that HER2 expression may be a more significant prognostic factor than PR loss in tumors with HER2 overexpression (HER2+) or may be associated with the results of trastuzumab treatment.

However, in tumors without HER2 overexpression, single HR positivity is a significant prognostic factor. The survival graph of ER + PR-tumors is between that of ER + PR+ tumors and ER-PR- tumors initially, but falls to as poor as TNBC at about the 10-year follow-up. Therefore, ER + PR-HER2- and TNBC tumors show no difference in terms of long-term survival. ER-PR + HER2- tumors show similar biological features to TNBC, including high Ki-67 level and high expression of EGFR (about 90%) and p53 (50%). Previous studies have shown incidence rate and clinicopathologic features, and ER-PR+ tumors have increased an incidence in premenopausal women and of an aggressive phenotype with higher tumor grade and HER2 overexpression [10,19,20]. Our results are consistent with those of previous studies. In our series, although there were only a few ER-PR + HER2- tumors, approximately 80% (43/53) of patients with ER-PR + HER2- tumors received chemotherapy and endocrine therapy. Nevertheless, the aggressive behavior of these tumors suggests that ER-PR+ tumors are very rare and represent a distinct biological subtype.

Our study is consistent with previous large studies demonstrating that single HR+ tumors have high expression of EGFR/HER2 and more aggressive features than ER + PR+ tumors. However, our results show that single HR positivity was not a significant prognostic factor in HER2+ breast cancer. Therefore, the aggressiveness of single HR+ tumors is not simply due to hyperactive growth factor signaling pathways. As in recent studies [7-9], our cases have shown that single HR+ tumors are associated with a high level of Ki-67 (≥14.0%), and, in multivariate analysis, Ki-67 was shown to have borderline significance (P = 0.068). When we additionally analyzed according to Ki-67 level, in cases with a high level of Ki-67 (≥14.0%), the differences among the four subtypes were still shown consistently. However, in patients with a low Ki-67 level (<14.0%), the prognosis of ER + PR- tumors was not different from that of ER + PR+ tumors. These results may suggest that PR is a significant prognostic factor in HR + HER2- tumors with a high level of Ki-67 expression, but not in HR + HER2+ tumors. These suggest that proliferation-related genes may be significantly associated with PR negativity. However, interestingly, ER-PR+ tumors have been shown to have the worst prognosis of the subtypes, regardless of Ki-67 level, suggesting that ER-PR+ tumors represent a distinct biological subtype.

This study had several limitations. It was a retrospective study, and adjuvant treatment was not determined on a randomized basis. Furthermore, we did not stratify patients according to treatment with tamoxifen or aromatase inhibitors. Although the use of aromatase inhibitors instead of selective estrogen receptor modulators improved the outcome of ER + PR− patients in the ATAC trial [21], the BIG 1-98 trial did not demonstrate a significant benefit of letrozole over tamoxifen in ER + PR− tumors [22]. In addition, at our center, most postmenopausal patients with HR+ tumors received aromatase inhibitor, excluding patients with contraindications or adverse effects.

Recent studies have emphasized the influence of PR, which provides highly significant stratification of ER+ breast cancer into luminal A and B types [7-9]. Prat et al. proposed that the IHC-based definition of luminal A tumors is HR+/HER2-/low Ki-67 (less than 14%), and high PR (more than 20%) [8] and Braun et al. also defined luminal B tumors by the presence of high-risk criteria (loss of PR expression or increased proliferation) [9]. Cancello et al. suggested that PR loss identifies luminal B breast cancer subgroups at higher risk of relapse and death, both with HER-2+ and HER-2- breast cancer [7]. The differences observed in HER2+ tumors in our study may be the result of differences in chemotherapy and trastuzumab treatment. In that study, about 30% of patients with ER + PR + HER2+ and ER + PR − HER2+ tumors received endocrine therapy alone, 65–70% received chemotherapy plus endocrine therapy as adjuvant treatments and about 1% in both the ER + PR + HER2+ and ER + PR − HER2+ subgroups received trastuzumab as an adjuvant therapy[7]. However, in our study, 89% of patients with HER2 overexpression received chemotherapy and 57.2% of patients received trastuzumab treatment.

## Conclusions

This study has identified clinically and biologically distinct features of single HR+ tumors (ER + PR- and ER-PR+) through comparison with both ER + PR+ tumors and ER-PR- tumors. These differences were significant in HER2- tumors, but not in HER2+ tumors. ER + PR-HER2- tumors and ER-PR + HER2- tumors have poorer survival than ER + PR + HER2- tumors and a similarly poor survival in comparison to ER-PR-HER2- tumors (TNBC). Clinical trials in addition to more advanced biological and molecular studies are necessary to identify the cause of aggressiveness in single HR+ tumors.

## Additional file

Additional file 1: Table S1. Clinicopathologic characteristics of patients with HER2-negative tumors. Table S2 Clinicopathologic characteristics of patients with HER2-positive tumors.

## Abbreviations

HR: Hormone receptor
ER: Estrogen receptor
PR: Progesterone receptor
DFS: Disease-free survival
OS: Overall survival
HER2: Human epidermal growth factor receptor 2
EGFR: Epidermal growth factor receptors
NG: Nuclear grade

## References

[1] Bardou V-J, Arpino G, Elledge RM, Osborne CK, Clark GM (2003). Progesterone Receptor Status Significantly Improves Outcome Prediction Over Estrogen Receptor Status Alone for Adjuvant Endocrine Therapy in Two Large Breast Cancer Databases. J Clin Oncol.

[2] Elledge RM, Green S, Pugh R, Allred DC, Clark GM, Hill J (2000). Estrogen receptor (ER) and progesterone receptor (PgR), by ligand-binding assay compared with ER, PgR and pS2, by immuno-histochemistry in predicting response to tamoxifen in metastatic breast cancer: a Southwest Oncology Group Study. Int J Cancer.

[3] Ravdin PM, Green S, Dorr TM, McGuire WL, Fabian C, Pugh RP (1992). Prognostic significance of progesterone receptor levels in estrogen receptor-positive patients with metastatic breast cancer treated with tamoxifen: results of a prospective Southwest Oncology Group study. J Clin Oncol.

[4] Arpino G, Weiss H, Lee AV, Schiff R, De Placido S, Osborne CK (2005). Estrogen Receptor–Positive, Progesterone Receptor–Negative Breast Cancer: Association With Growth Factor Receptor Expression and Tamoxifen Resistance. J Natl Cancer Inst.

[5] Punglia RS, Kuntz KM, Winer EP, Weeks JC, Burstein HJ (2006). The impact of tumor progesterone receptor status on optimal adjuvant endocrine therapy for postmenopausal patients with early-stage breast cancer. Cancer.

[6] Stuart-Harris R, Shadbolt B, Palmqvist C, Chaudri Ross HA (2009). The prognostic significance of single hormone receptor positive metastatic breast cancer: an analysis of three randomised phase III trials of aromatase inhibitors. Breast.

[7] Cancello G, Maisonneuve P, Rotmensz N, Viale G, Mastropasqua MG, Pruneri G (2013). Progesterone receptor loss identifies Luminal B breast cancer subgroups at higher risk of relapse. Ann Oncol.

[8] Prat A, Cheang MC, Martin M, Parker JS, Carrasco E, Caballero R (2013). Prognostic significance of progesterone receptor-positive tumor cells within immunohistochemically defined luminal A breast cancer. J Clin Oncol.

[9] Braun L, Mietzsch F, Seibold P, Schneeweiss A, Schirmacher P, Chang-Claude J (2013). Intrinsic breast cancer subtypes defined by estrogen receptor signalling-prognostic relevance of progesterone receptor loss. Mod Pathol.

[10] Rakha EA, El-Sayed ME, Green AR, Paish EC, Powe DG, Gee J (2007). Biologic and Clinical Characteristics of Breast Cancer With Single Hormone Receptor–Positive Phenotype. J Clin Oncol.

[11] Cui X, Schiff R, Arpino G, Osborne CK, Lee AV (2005). Biology of progesterone receptor loss in breast cancer and its implications for endocrine therapy. J Clin Oncol.

[12] Rhodes A, Jasani B (2009). The oestrogen receptor-negative/progesterone receptor-positive breast tumour: a biological entity or a technical artefact?. J Clin Pathol.

[13] De Maeyer L, Van Limbergen E, De Nys K, Moerman P, Pochet N, Hendrickx W (2008). Does estrogen receptor negative/progesterone receptor positive breast carcinoma exist?. J Clin Oncol.

[14] Keshgegian AA, Cnaan A (1996). Estrogen receptor-negative, progesterone receptor-positive breast carcinoma: poor clinical outcome. Arch Pathol Lab Med.

[15] Sundblad AS, Caprarulo L (1996). Immunohistochemical characteristics of mammary carcinomas with estrogen-negative and progesterone-positive receptors. Medicina (B Aires).

[16] Dunnwald LK, Rossing MA, Li CI (2007). Hormone receptor status, tumor characteristics, and prognosis: a prospective cohort of breast cancer patients. Breast Cancer Res.

[17] Goldhirsch A, Ingle JN, Gelber RD, Coates AS, Thürlimann B, Senn H-J (2009). Thresholds for therapies: highlights of the St Gallen International Expert Consensus on the Primary Therapy of Early Breast Cancer 2009. Ann Oncol.

[18] Colomer R, Beltran M, Dorcas J, Cortes-Funes H, Hornedo J, Valentin V (2005). It Is Not Time to Stop Progesterone Receptor Testing in Breast Cancer. J Clin Oncol.

[19] Nadji M, Gomez-Fernandez C, Ganjei-Azar P, Morales AR (2005). Immunohistochemistry of estrogen and progesterone receptors reconsidered: experience with 5,993 breast cancers. Am J Clin Pathol.

[20] Huang HJ, Neven P, Drijkoningen M, Paridaens R, Wildiers H, Van Limbergen E (2005). Association between tumour characteristics and HER-2/neu by immunohistochemistry in 1362 women with primary operable breast cancer. J Clin Pathol.

[21] Howell A, Cuzick J, Baum M, Buzdar A, Dowsett M, Forbes JF (2005). Results of the ATAC (Arimidex, Tamoxifen, Alone or in Combination) trial after completion of 5 years’ adjuvant treatment for breast cancer. Lancet.

[22] Viale G, Regan MM, Maiorano E, Mastropasqua MG, Dell'Orto P, Rasmussen BB (2007). Prognostic and predictive value of centrally reviewed expression of estrogen and progesterone receptors in a randomized trial comparing letrozole and tamoxifen adjuvant therapy for postmenopausal early breast cancer: BIG 1-98. J Clin Oncol.

