# Supplementary material for: Poor prognosis of single hormone receptor- positive breast cancer: similar outcome as triple-negative breast cancer
Source: BMC Cancer. 2015 Mar 18;15:138. doi: 10.1186/s12885-015-1121-4 (PMC4396721; doi:10.1186/s12885-015-1121-4)
Supplement: Additional file 1: Table S1. — Clinicopathologic characteristics of patients with HER2-negative tumors. Table S2 Clinicopathologic characteristics of patients with HER2-positive tumors. [file 12885_2015_1121_MOESM1_ESM.doc]

Table S1. Clinicopathologic characteristics of patients with HER2-negative tumors

|  | ER+PR+ (N=518) | | ER-PR+ (N=30) | | ER-PR- (N=671) | | ER+ PR- (N=159) | |  |
| --- | --- | --- | --- | --- | --- | --- | --- | --- | --- |
| Age, median (range) | 47 | (23-77) | 49.5 | (31-72) | 52 | (27-85) | 53 | (91-75) |  |
| Menopause | |  |  |  |  |  |  |  |  |
| Postmenopause | 173 | 33.80% | 12 | 41.40% | 384 | 58.10% | 102 | 65.00% | <0.001 |
| Premenopause | 339 | 66.20% | 17 | 58.60% | 277 | 41.90% | 55 | 35.00% |  |
| Unknown | 6 |  | 1 |  | 10 |  | 2 |  |  |
| Operation | |  |  |  |  |  |  |  | 0.001 |
| MRM | 201 | 38.80% | 21 | 70.00% | 312 | 46.50% | 67 | 42.10% |  |
| BCS | 317 | 61.20% | 9 | 30.00% | 359 | 53.50% | 92 | 57.90% |  |
| pT |  |  |  |  |  |  |  |  |  |
| T1 | 292 | 56.40% | 18 | 60.00% | 365 | 54.40% | 82 | 51.60% | 0.726 |
| T2 | 202 | 39.00% | 12 | 40.00% | 276 | 41.10% | 69 | 43.40% |  |
| T3 | 24 | 4.60% | 0 | 0.00% | 29 | 4.30% | 7 | 4.40% |  |
| T4 | 0 | 0.00% | 0 | 0.00% | 1 | 0.10% | 1 | 0.60% |  |
| pN |  |  |  |  |  |  |  |  |  |
| N0 | 282 | 54.40% | 14 | 46.70% | 385 | 57.40% | 94 | 59.10% | 0.661 |
| N1 | 146 | 28.20% | 12 | 40.00% | 183 | 27.30% | 43 | 27.00% |  |
| N2 | 51 | 9.80% | 2 | 6.70% | 69 | 10.30% | 13 | 8.20% |  |
| N3 | 39 | 7.50% | 2 | 6.70% | 34 | 5.10% | 9 | 5.70% |  |
| Stage |  |  |  |  |  |  |  |  |  |
| I | 199 | 38.40% | 10 | 33.30% | 254 | 37.90% | 59 | 37.10% | 0.869 |
| IIA | 150 | 29.00% | 11 | 36.70% | 206 | 30.70% | 53 | 33.30% |  |
| IIB | 70 | 13.50% | 5 | 16.70% | 95 | 14.20% | 21 | 13.20% |  |
| IIIA | 60 | 11.60% | 2 | 6.70% | 81 | 12.10% | 16 | 10.10% |  |
| IIIB | 0 | 0.00% | 0 | 0.00% | 1 | 0.10% | 1 | 0.60% |  |
| IIIC | 39 | 7.50% | 2 | 6.70% | 34 | 5.10% | 9 | 5.70% |  |
| Nuclear Grade | |  |  |  |  |  |  |  | <0.001 |
| I | 13 | 2.50% | 0 | 0.00% | 2 | 0.30% | 3 | 1.90% |  |
| II | 212 | 41.00% | 8 | 26.70% | 167 | 24.90% | 57 | 35.80% |  |
| III | 292 | 56.50% | 22 | 73.30% | 501 | 74.80% | 99 | 62.30% |  |
| unknown | 1 |  |  |  | 1 |  |  |  |  |
| Ki-67 |  |  |  |  |  |  |  |  | 0.041 |
| ≥ 14.0 | 423 | 88.30% | 18 | 94.70% | 513 | 92.90% | 119 | 87.50% |  |
| < 14.0 | 56 | 11.70% | 1 | 5.30% | 39 | 7.10% | 17 | 12.50% |  |
| Unknown | 39 |  | 11 |  | 119 |  | 23 |  |  |
| p53 |  |  |  |  |  |  |  |  | <0.001 |
| Positive | 229 | 44.40% | 19 | 63.30% | 397 | 59.30% | 75 | 47.50% |  |
| Negative | 287 | 55.60% | 11 | 36.70% | 273 | 40.70% | 83 | 52.50% |  |
| Unknown | 2 |  |  |  | 1 |  | 1 |  |  |
| Chemotherapy | |  |  |  |  |  |  |  | 0.37 |
| Yes | 454 | 90.80% | 26 | 86.70% | 573 | 88.40% | 133 | 86.40% | <0.001 |
| No | 46 | 9.20% | 4 | 13.30% | 75 | 11.60% | 21 | 13.60% |  |
| Unknown | 18 |  |  |  | 23 |  | 5 |  |  |
| Radiotherapy | |  |  |  |  |  |  |  |  |
| Yes | 357 | 71.30% | 11 | 36.70% | 413 | 64.00% | 103 | 66.90% | <0.001 |
| No | 144 | 28.70% | 19 | 63.30% | 232 | 36.00% | 51 | 33.10% |  |
| Unknown | 501 | 1700.00% | 30 | 0.00% | 645 | 2600.00% | 154 | 500.00% |  |
| Endocrine Therapy | |  |  |  |  |  |  |  |  |
| Yes | 479 | 97.20% | 25 | 86.20% | 1 | 0.20% | 143 | 93.50% | <0.001 |
| No | 14 | 2.80% | 4 | 13.80% | 646 | 99.80% | 10 | 6.50% |  |
| Unknown | 493 | 2500.00% | 29 | 100.00% | 647 | 2400.00% | 153 | 600.00% |  |
| Trastuzumab |  |  |  |  |  |  |  |  |  |
| Yes | 333 | 64.30% | 13 | 43.30% | 360 | 53.70% | 84 | 52.80% | <0.001 |
| No | 185 | 35.70% | 17 | 56.70% | 311 | 46.30% | 75 | 47.20% |  |
| Unknown | 518 | 0.00% | 30 | 0.00% | 671 | 0.00% | 159 | 0.00% |  |

Table S2. Clinicopathologic characteristics of patients with HER2-positive tumors

|  | ER+PR+ (n=4018) | | ER-PR+ (n=57) | | ER-PR- (n=1058) | | ER+ PR-(n=302) | |  |
| --- | --- | --- | --- | --- | --- | --- | --- | --- | --- |
| Age, median (range) | 47 | (21-90) | 47 | (23-71) | 48 | (21-85) | 55 | (27-84) |  |
| Menopause |  |  |  |  |  |  |  |  |  |
| Postmenopause | 1284 | -32.3% | 22 | 39.3% | 437 | 42.1% | 206 | 68.9% | <0.001 |
| Premenopause | 2690 | 67.7% | 34 | 60.7% | 602 | 57.9% | 93 | 31.1% |  |
| Unknown | 44 |  | 1 |  | 19 |  | 3 |  |  |
| Operation |  |  |  |  |  |  |  |  |  |
| MRM | 1105 | 27.5% | 17 | 29.8% | 258 | 24.4% | 95 | 31.5% | 0.061 |
| BCS | 2913 | 72.5% | 40 | 70.2% | 800 | 75.6% | 207 | 68.5% |  |
| pT |  |  |  |  |  |  |  |  |  |
| T1 | 2620 | 65.2% | 29 | 50.9% | 481 | 45.5% | 193 | 63.9% | <0.001 |
| T2 | 1234 | 30.7% | 25 | 43.9% | 545 | 51.5% | 106 | 35.1% |  |
| T3 | 154 | 3.8% | 3 | 5.3% | 32 | 3.0% | 3 | 1.0% |  |
| T4 | 10 | 0.2% | 0 | 0.0% | 0 | 0.0% | 0 | 0.0% |  |
| pN |  |  |  |  |  |  |  |  |  |
| N0 | 2358 | 58.7% | 32 | 56.1% | 700 | 66.2% | 194 | 64.2% | <0.003 |
| N1 | 1192 | 29.7% | 16 | 28.1% | 255 | 24.1% | 82 | 27.2% |  |
| N2 | 308 | 7.7% | 6 | 10.5% | 62 | 5.9% | 19 | 6.3% |  |
| N3 | 160 | 4.0% | 3 | 5.3% | 41 | 3.9% | 7 | 2.3% |  |
| Stage |  |  |  |  |  |  |  |  |  |
| I | 1898 | 47.2% | 20 | 35.1% | 365 | 34.5% | 142 | 47.0% | <0.001 |
| IIA | 1087 | 27.1% | 19 | 33.3% | 418 | 39.5% | 101 | 33.4% |  |
| IIB | 518 | 12.9% | 8 | 14.0% | 166 | 15.7% | 31 | 10.3% |  |
| IIIA | 347 | 8.6% | 7 | 12.3% | 68 | 6.4% | 21 | 7.0% |  |
| IIIB | 8 | 0.2% | 0 | 0.0% | 0 | 0.0% | 0 | 0.0% |  |
| IIIC | 160 | 4.0% | 3 | 5.3% | 41 | 3.9% | 7 | 2.3% |  |
| Nuclear Grade | |  |  |  |  |  |  |  |  |
| I | 935 | 23.4% | 1 | 1.8% | 10 | 0.9% | 58 | 19.4% | <0.001 |
| II | 2376 | 59.3% | 16 | 28.6% | 183 | 17.3% | 138 | 46.2% |  |
| III | 693 | 17.3% | 39 | 69.6% | 862 | 81.7% | 103 | 34.4% |  |
| Unknown | 14 |  | 1 |  | 3 |  | 3 |  |  |
| Ki-67 |  |  |  |  |  |  |  |  |  |
| ≥ 14.0 | 1748 | 49.3% | 38 | 90.5% | 811 | 94.6% | 166 | 69.5% | <0.001 |
| < 14.0 | 1800 | 50.7% | 4 | 9.5% | 46 | 5.4% | 73 | 30.5% |  |
| Unknown | 470 |  | 15 |  | 201 |  | 63 |  |  |
| p53 |  |  |  |  |  |  |  |  |  |
| Positive | 747 | 18.7% | 28 | 50.0% | 610 | 58.1% | 86 | 29.1% | <0.001 |
| Negative | 3253 | 81.3% | 28 | 50.0% | 440 | 41.9% | 210 | 70.9% |  |
| Unknown | 18 |  | 1 |  | 8 |  | 6 |  |  |
| Chemotherapy | |  |  |  |  |  |  |  |  |
| Yes | 2600 | 66.0% | 49 | 90.7% | 980 | 94.5% | 202 | 68.7% | <0.001 |
| No | 1341 | 34.0% | 5 | 9.3% | 57 | 5.5% | 92 | 31.3% |  |
| Unknown | 77 |  | 3 |  | 21 |  | 8 |  |  |
| Radiotherapy | |  |  |  |  |  |  |  |  |
| Yes | 3122 | 79.4% | 43 | 81.1% | 829 | 80.5% | 218 | 73.4% | 0 |
| No | 812 | 20.6% | 10 | 18.9% | 201 | 19.5% | 79 | 26.6% |  |
| Unknown | 84 |  | 4 |  | 28 |  | 5 |  |  |
| Endocrine Therapy | |  |  |  |  |  |  |  |  |
| Yes | 3902 | 99.5% | 47 | 88.7% | 1 | 0.1% | 291 | 98.6% | <0.001 |
| No | 20 | 0.5% | 6 | 11.3% | 1029 | 99.9% | 4 | 1.4% |  |
| Unknown | 96 |  | 4 |  | 28 |  | 7 |  |  |
